# Long-term physical functioning and health-related outcomes in survivors of intensive care

**DOI:** 10.1186/cc13242

**Published:** 2014-03-17

**Authors:** K Solverson, C Doig

**Affiliations:** 1 University of Calgary, Canada

## Introduction

The long-term impact of critical illness on survivors' physical and mental health remains unknown. Our aim was to create a follow-up clinic for survivors of critical illness in order to quantitatively examine muscle weakness, physical functioning and mental health and relate these findings to health-related quality of life (HRQL) and ICU risk factors.

## Methods

Study patients were selected from a 24-bed multidisciplinary ICU. Fifty-six patients who were ≥18 years old, stayed longer than 4 days in the ICU and did not have an acute brain injury were followed-up at 2 and 4 months post hospital discharge. Peripheral muscle strength (grip, triceps, biceps, hamstrings, quadriceps and dorsiflexors) and physical functioning were objectively assessed using hand-held dynamometry and the six-minute walk test (6MWT); both were compared with age/ sex normative data. HRQL and mental health were assessed using the Short Form-36 (SF-36), EuroQol-5D (EQ-5D) and Hospital Anxiety and Depression Scale.

## Results

The median age of patients was 60 years, 68% were admitted for respiratory illnesses, they were severely ill (median APACHE II, 19) and had long ICU lengths of stay (median, 11 days). Muscle strength was significantly reduced when compared with normative data in all muscle groups at the 2-month and 4-month visits (2-month average indexed overall strength, 72%, *P *< 0.05). The median distance walked during the 6MWT was 382 m at 2 months (median percent predicted, 72%) and it did not significantly change at the 4-month visit. Reduced peripheral muscle strength was significantly correlated with lower distance walked during the 6MWT. Reported HRQL by the SF-36 was below national averages at both 2-month and 4-month visits (2-month physical composite score (PCS) 36.2, mental composite score 48.1; 50 = national average). Reduced muscle strength was associated with low scores on the SF-36 physical function and general health domains. Performance on the 6MWT correlated with the SF-36 including the PCS (*P *= 0.001). Screening positive for anxiety was associated with both poor 6MWT performance and reporting dysfunction on the EQ- 5D domains. ICU/hospital length of stay, number of days ventilated, severity of illness and organ dysfunction were not found to be predictive of muscle strength or physical functioning.

## Conclusion

Our study gives qualitative evidence that survivors of critical illness have reduced muscle strength, physical functioning and HRQL after hospital discharge. Also, we have shown muscle weakness is predictive of overall physical functioning, which in turn impacted HRQL and mental health. No ICU risk factors were identified that predicted deficits in muscle strength or physical functioning.

